# Supplementary material for: Introduced Spiders in Panama: Species Distributions and New Records
Source: Biology (Basel). 2024 Dec 24;14(1):4. doi: 10.3390/biology14010004 (PMC11762183; doi:10.3390/biology14010004)
Supplement: Supplementary file 1 [file biology-14-00004-s001.zip › biology-3359074-supplementary.pdf]

Supplementary material for the manuscript ‘Introduced spiders in Panama: species distributions and new records’

Table S1. Results of searches carried in Web of Sciences. The term ‘Search’ indicates the initial general search, and all the searches were refined with the term ‘Panama’.

	Terms			
	Invasive species	Invasive spider	Especie invasora	Especie exotica
Refined search	Panama	Panama	Panama	Panama
# of documents	271	1	1	1
# of documents about spiders	1	0	0	0
# of documents reporting new invasive spiders	**0**	**0**	**0**	**0**

Table S2. Coordinates of collected individuals of invasive spiders in Panama.

**species**	**latitude**	**longitude**
Cyrtophora citricola	8.983662	-79.530094
Cyrtophora citricola	8.9943897	-79.543258
Cyrtophora citricola	8.886773	-78.289506
Cyrtophora citricola	8.26568534	-82.291723
Cyrtophora citricola	8.25801375	-82.317095
Cyrtophora citricola	9.27305352	-82.1434
Cyrtophora citricola	7.45116	-80.08122
Cyrtophora citricola	7.803063	-80.258949
Cyrtophora citricola	7.5344815	-80.02606
Cyrtophora citricola	8.5812721	-80.399286
Cyrtophora citricola	7.496501	-81.221415
Cyrtophora citricola	7.634114	-81.258222
Cyrtophora citricola	9.117138	-79.700164
Labahitha marginata	8.3986691	-80.26309
Labahitha marginata	7.75575	-77.68414
Labahitha marginata	8.252926	-78.164657
Labahitha marginata	8.836209	-78.267144
Labahitha marginata	8.25801375	-82.317095
Labahitha marginata	7.415291	-80.172314
Labahitha marginata	9.119082	-79.693755
Labahitha marginata	7.63778	-81.703812
Labahitha marginata	9.1513019	-79.077962
Labahitha marginata	9.1088068	-79.45504
Physocyclus globosus	8.9861377	-79.532633
Physocyclus globosus	8.96238	-79.54379
Physocyclus globosus	8.252926	-78.164657
Physocyclus globosus	9.0017097	-79.583493
Physocyclus globosus	9.1513019	-79.077962
Theridion melanostictum	8.960801	-79.544377
Theridion melanostictum	9.117126	-79.692759
Theridion melanostictum	9.0017097	-79.583493

Micropholcus fauroti	8.99392	-79.53542
Micropholcus fauroti	7.777057	-80.270994
Micropholcus fauroti	9.1066778	-79.296873
Micropholcus fauroti	8.659255	-79.874349
Cithaeron praedonius	7.777057	-80.270994
Latrodectus geometricus	8.9861377	-79.532633
Hasarius adansoni	8.9861377	-79.532633
Neoscona nautica	9.066345	-79.388674
Crossopriza lyoni	9.066345	-79.388674

Table S3. Summary of presence / absence (1 and 0, respectively) of invasive spider species per regions of Panama. BC: Bocas del Toro, CH: Chiriquí, C: Colón, CO: Coclé, D: Darién, H: Herrera, GY: Guna Yala (indigenous territory), LS: Los Santos, PA: Panamá, PO: Panamá Oeste, V: Veraguas. Lit: Literature review, Inat: Inaturalist, sampled: sampled in the field by this study, museum: revision of museum collection. Bold X indicates new reports found by this study. See Table 1 in main document for details on species authorities.

														This study			
**Species**	**BC**	**CH**	**C**	**CO**	**D**	**H**	**GY**	**LS**	**PA**	**PO**	**V**	**Total**	**Lit**	**Inat**	**Gbif**	**sampled**	**museum**
Cyrtophora citricola	1	1	1	1	1	1	1	1	1	1	1	**11**		**x**		**x**	
Heteropoda venatoria	0	1	1	1	1	0	1	0	1	1	1	**8**	x	x			
Labahitha marginata	0	1	1	1	1	0	0	1	1	0	1	**7**	x			x	
Physocyclus globosus	0	1	1	1	1	0	0	0	1	1	1	**7**	x			x	
Neoscona nautica	1	1	0	0	0	0	0	0	1	1	1	**5**	x				
Triaeris stenaspis	1	1	1	0	0	0	0	0	1	1	0	**5**	x				
Hasarius adansoni	0	1	1	1	0	0	0	0	1	0	1	**5**	x	x		x	x
Tetragnatha nitens	0	1	1	1	0	0	0	0	1	1	0	**5**	x				x
Ischnothyreus peltifer	0	0	1	0	0	0	0	1	1	1	0	**4**	x				x
Menemerus bivittatus	0	1	0	1	0	0	0	0	1	1	0	**4**	x	x		x	x
Plexippus paykulli	0	0	1	0	0	0	0	0	1	1	1	**4**	x	x		x	x
Meotipa pulcherrima	0	1	1	0	0	0	0	0	0	1	1	**4**	x				
Cithaeron praedonius	0	0	0	0	0	0	0	1	1	0	1	**3**	x	x		x	
Brignolia parumpunctata	0	0	1	0	0	0	0	0	1	1	0	**3**	x				
Opopaea apicalis	0	0	1	0	0	0	0	0	1	1	0	**3**	x				
Opopaea deserticola	0	0	1	0	0	0	0	0	1	1	0	**3**	x				x
Micropholcus fauroti	0	0	0	0	0	0	0	1	1	1	0	**3**	x			x	
Latrodectus mactans	0	1	0	0	0	0	0	0	0	1	1	**3**	x				
Steatoda erigoniformis	0	0	1	0	0	0	0	0	1	0	1	**3**	x				
Theridion melanostictum	0	0	1	0	0	0	0	0	1	1	0	**3**			**x**	**x**	
Gea heptagon	0	0	0	0	0	0	0	0	1	1	0	**2**	x				x
Ischnothyreus velox	0	0	1	0	0	0	0	0	1	0	0	**2**	x				
Xestaspis parmata	0	0	0	0	0	0	0	0	1	1	0	**2**	x		x		
Latrodectus geometricus	0	0	1	0	0	0	0	0	1	0	0	**2**	x			x	x
Steatoda grossa	0	1	0	0	0	0	0	0	0	0	1	**2**		**x**			
Neoscona adianta	0	1	0	0	0	0	0	0	0	0	0	**1**			**x**		
Theotima minutissima	0	0	0	0	0	0	0	0	0	1	0	**1**	x				
Artema atlanta	0	0	1	0	0	0	0	0	0	0	0	**1**	x				
Crossopriza lyoni	0	0	0	0	0	0	0	0	1	0	0	**1**				**x**	
Tetragnatha vermiformis	0	0	0	0	0	0	0	0	0	1	0	1	x				
Parasteatoda tepidariorum	0	1	0	0	0	0	0	0	0	0	0	1		**x**	**x**		
	**3**	**14**	**18**	**7**	**4**	**1**	**2**	**5**	**23**	**20**	**12**						

Table S4. Details on some unconfirmed exotic spider species in Panama.

Species	Details
Neoscona adianta (Walckenaer, 1802)	Although there is a specimen identified as coming from Panama and deposited in the Field Museum of Natural History [78], this species has not been reported as cosmopolitan or introduced in other countries, remaining within its natural range. Although it has shown an expansion in recent years [12,31,115], it is possible that this record corresponds to a misidentification, which will be confirmed after a detailed review of the specimen.
